# Potential Novel Biomarkers for Mastitis Diagnosis in Sheep

**DOI:** 10.3390/ani11102783

**Published:** 2021-09-24

**Authors:** Kacper Libera, Kacper Konieczny, Julia Grabska, Sebastian Smulski, Izabela Szczerbal, Małgorzata Szumacher-Strabel, Małgorzata Pomorska-Mól

**Affiliations:** 1 Department of Preclinical Sciences and Infectious Diseases, Poznan University of Life Sciences, Wołyńska 35, 60-637 Poznań, Poland; kacper.libera@up.poznan.pl (K.L.); julsgrabska@gmail.com (J.G.); 2 Department of Internal Diseases and Diagnostics, Poznan University of Life Sciences, Wołyńska 35, 60-637 Poznań, Poland; kacper.konieczny@up.poznan.pl (K.K.); sebastian.smulski@up.poznan.pl (S.S.); 3 Department of Genetics and Animal Breeding, Poznan University of Life Sciences, Wołyńska 33, 60-637 Poznań, Poland; izabela.szczerbal@up.poznan.pl; 4 Department of Animal Nutrition, Poznan University of Life Sciences, Wołyńska 33, 60-637 Poznań, Poland; malgorzata.szumacher@up.poznan.pl

**Keywords:** ewe, udder inflammation, ovine, milk quality

## Abstract

**Simple Summary:**

Inflammation of the mammary gland (mastitis) is an important disease of dairy sheep. Mastitis management depends mainly on the diagnosis. Conventional diagnostic methods including somatic cell count, California Mastitis Test, and microbial culture have limitations. Therefore researchers are looking for new diagnostic biomarkers of mastitis including specific proteins produced by the liver in case of disease (acute phase proteins), unique genetic sequences (miRNAs), or antimicrobial peptides produced by immune cells during inflammation (cathelicidines).

**Abstract:**

This review aims to characterize promising novel markers of ovine mastitis. Mastitis is considered as one of the primary factors for premature culling in dairy sheep and has noticeable financial, productional, and animal welfare-related implications. Furthermore, clinical, and subclinical mammary infections negatively affect milk yield and alter the milk composition, thereby leading to lowered quality of dairy products. It is, therefore, crucial to control and prevent mastitis through proper diagnosis, treatment or culling, and appropriate udder health management particularly at the end of the lactation period. The clinical form of mastitis is characterized by abnormalities in milk and mammary gland tissue alteration or systemic symptoms consequently causing minor diagnostic difficulties. However, to identify ewes with subclinical mastitis, laboratory diagnostics is crucial. Mastitis control is primarily dependent on determining somatic cell count (SCC) and the California Mastitis Test (CMT), which aim to detect the quantity of cells in the milk sample. The other useful diagnostic tool is microbial culture, which complements SCC and CMT. However, all mentioned diagnostic methods have their limitations and therefore novel biomarkers of ovine subclinical mastitis are highly desired. These sensitive indicators include acute-phase proteins, miRNA, and cathelicidins measurements, which could be determined in ovine serum and/or milk and in the future may become useful in early mastitis diagnostics as well as a preventive tool. This may contribute to increased detection of ovine mammary gland inflammation in sheep, especially in subclinical form, and consequently improves milk quality and quantity.

## 1. Introduction

Mammary gland inflammation (mastitis) is one of the most costly and severe diseases in the dairy industry [1]. Negative impact does not only refer to economic reasons but also significantly contributes to animals’ health and consequently their welfare. Another important perspective is the food safety (food-borne diseases) and quality of dairy products (such as cheese) since milk from affected animals may contain pathogenic bacteria and has altered composition undesired by the dairy industry [2,3]. From a global point of view, the most important dairy species are cattle, producing over 80% of world milk production [4] followed by buffaloes with 15%, goats with 2% (accounts for about 1000 million head, of which 20% intended for dairy production) and sheep with 1% (accounts for almost 1200 million head, of which 25% intended for dairy production); camels provide 0.5% and 1.5% comes from other dairy species [4]. Consequently, mastitis in cattle is a well-recognized problem, to which researchers and bovine practitioners all around the world pay special attention. Nevertheless, in some countries due to climate and/or traditional or historical reasons milk is derived from other than cattle dairy species, for example, from small ruminants [5]. Goats and sheep are often kept in an environment with scarce grazing and unfavorable climatic conditions. In some countries, they are considered as dairy animals of the poor because of the lower capital investment and low production costs required [6]. They are also characterized by rapid generation turnover (and thus earlier milk production compared with other dairy animals), short pregnancies, and milk supply in quantities that are suitable for immediate household consumption (thereby reducing problems of milk storage and marketing) [5]. However, at the same time, small ruminants become more and more popular in highly developed countries since they perfectly fit into the conception of organic farming or are kept as pets, in particular in suburban areas [7,8]. Although sheep and goats are not demanding animals, they can provide products of great quality. Ewe’s milk contains higher levels of total solids (protein and fat) and more major nutrients than goat and cow milk. To compare, sheep milk contains 5.5 ± 1.1 g/100 g of protein and 5.9 ± 0.3 g/100 g of fat, while cow milk contains 3.4 ± 0.1 g/100 g and 3.3 ± 0.2 g/100 g of these solids, respectively [9]. Consequently, ewe’s milk is characterized by excellent cheese-making properties, thus is consumed rarely in liquid form. Apart from that, there is one compound in ruminants’ milk—conjugated linoleic acid (CLA), the most abundant in sheep milk, that may have far-reaching, positive effects on milk consumption [10]. CLA has been shown to have numerous potential benefits for human health, including potent cancer-fighting properties [10]. Sheep milk-producing farms represent a significant part of the agrarian economies in many countries, especially those bordering the Mediterranean Sea and in the Middle East [9]. Moreover, it is important to highlight that dairy sheep are suitable for organic agriculture, involving a long period of grazing, great care for animal welfare, and reduced use of antibiotics and hormones. The bio (organic) products derived from organic farming are more and more popular in highly developed countries and this trend is expected to continue [11].

## 2. Methodological Approach in the Identification of References

The guidelines and the procedures as detailed by the Preferred Reporting Items for Systematic Reviews and Meta-Analysis (PRISMA) [12] were followed for drafting this review. Original research and review articles reporting mastitis in sheep until 15 August 2021 were searched through PubMed and Google Scholar databases. The search terms including “mastitis in sheep” or “ovine mastitis” or “biomarkers of mastitis” or “acute phase proteins in sheep” or “proteomics in sheep” or “genetic markers of mastitis in sheep” were entered one by one in PubMed and Google Scholar databases to identify all full-text research/review publications that cover mastitis in sheep (in particular ovine mastitis) thoroughly investigated for inclusion. Occasionally, the full-text articles were also requested from the authors, if the full-text article was not available online. Search results yielding articles in a language other than English were omitted from the analysis.

## 3. Mastitis in Dairy Sheep

Sheep are believed to be one of the first domesticated species and probably the problem of udder inflammation has been present since then [1]. Regarding mastitis in sheep the literature reports individual milk yield losses of 2.6–43.1% [2], being modulated by several factors including infection severity, production level, causal agents, and unilateral or bilateral infection. Mastitis not only negatively affects milk yield but also alters milk quality [7]. The impairment of physical and chemical characteristics due to decreased udder health status is responsible for the negative effect of increased somatic cell count on the coagulation properties of milk, the curd yield, and the quality of cheese [2], which does not allow producers to meet the quality standards required by consumers, industry and public health organizations [13]. A low ratio of casein to protein in high bulk tank somatic cell count (BTSCC) milk enhances the extension of the rennet coagulation time and curd firming time because there are more serum proteins, and the stability of casein micelles are reduced as a result of hydrolysis. Those changes, in turn, led to poor syneresis, lower cheese yield, increased moisture content, and lower fat and protein content in cheese [2]. 

Another important issue is public health in terms of consuming cheese made from infected milk, in particular, some traditional kinds of cheeses without milk pasteurization. Globally only 25% of sheep are intended for dairy production [14]. In many countries, most sheep are kept for the production of meat and therefore most studies focus on symptoms of mastitis occurring in ewes that are nursing lambs. In these flocks, only severe clinical mastitis is likely to be observed and diagnosed. According to Ruegg [15], this lack of emphasis on milking ewes has led to an over-emphasis on the occurrence of clinical mastitis and a lack of appreciation for subclinical mastitis. Clinical mastitis (CM) typically occurs in <5% of lactating ewes, but subclinical mastitis (SM) may occur in 15–30% of animals [15]. The information regarding mastitis prevalence in different management systems is given in Table 1. Among the etiologic agents, the most prevalent are Coagulase-negative staphylococci (CNS), *Corynebacterium* sp., while *Streptococcus* spp., *Enterobacteriaceae*, *Pseudomonas aeruginosa*, *Mannheimia haemolytica*, *Corynebacterium* spp., and fungi can also cause mastitis in sheep, but are observed at relatively lower rates [1]. Mastitis is considered one of the most significant reasons for premature culling in dairy sheep in the United Kingdom [16]. In the United States, udder-health issues account for about 14% of ewes culled each year [17]. There is no general consensus about the prevalence of mastitis in sheep of different breeds and from various areas. It has been reported that the culling of ewes resulting from clinical mastitis episodes can reach up to 70% [18] or even 90% [1]. Therefore, proper diagnosis is a critical aspect of preventing mastitis and its economic consequences. A significant role in mastitis control in the flock seems to have the udder health management at the end of the lactation period when the mammary gland is particularly prone to infections [19] and immediate or delayed culling. The elimination of existing subclinical infections relies on the intramammary application of antibiotics at drying-off and removing ewes affected by acute or chronic mastitis from the flock until culling or complete recovery [20]. Nevertheless, to identify the ewes with SM, laboratory diagnostics is crucial.

## 4. Conventional Approach to Mastitis Diagnosis

Laboratory diagnostics play an important role in the improvement of production efficiency and the control of diseases including mastitis [25]. Mastitis cases might be classified as clinical or subclinical. The clinical form is characterized by abnormalities in milk (i.e., presence of blood, pus, color change, or lumps), palpable possible mammary gland tissue alteration, or systemic symptoms (e.g., fever, loss of appetite) [3]. On the other hand, the subclinical form involves the mobilization of inflammatory cells to the udder, increasing the somatic cell count (SCC), but without alterations in gland tissue or milk aspect, thus are hard to identify under field conditions [26]. One of the on-the-spot tests such as the California Mastitis Test (CMT) can help to diagnose SM in ewes, but unlike in dairy cattle, the CMT is infrequently used to detect increases in bulk milk somatic cell counts, although it may have some application in dairy sheep. CMT has some limitations because it was originally developed for cows, and also due to its subjective nature, which can lead to inaccuracies in the interpretation of the results [25,26].

Likewise, there is no consensus in the literature regarding a cut-off value of SCC in sheep milk [25,27]. Moreover, even if the SCC in milk is considered a standard indicator of SM in cows it may not be a specific sign of the inflammatory status of the mammary gland in sheep, due to variability due to numerous factors other than intramammary infections including age, breed, management system, physiological stage of the animal (lactation stage, dry period), season, numbers of lambs born, and other factors [28]. Individual SCC are not commonly used in sheep to detect and treat subclinical mastitis.

Nevertheless, many authors have attempted to set a cut-off value for the somatic cell count in ewe’s milk; however, the data are still not consistent. Albenzio et al. [27] suggested that SCC > 300,000 cells/mL results in decreased milk production by the mammary gland. Other authors reported that the udder is considered healthy when the number of somatic cells does not exceed 250,000 cells/mL [29,30,31]. While Świderek et al. [32] stated that fluctuations of somatic cells in ewe’s milk up to 200,000 cells/mL are normal and only above this value are considered as the possible threshold for subclinical mastitis. In contrast, Miglio et al. [28] suggested that uncertain subclinical mastitis occurs when bacteriological testing is positive, or SCC > 500,000 cells/mL (non-specific-SM) in milk samples. Spanu et al. [33] presented that in ewes with 3 or more monthly SCC ≥ 400,000 cells/mL, detection of mastitis pathogens in their milk was 5.6–7.5 times more likely, compared to ewes with SCC below this threshold. According to Kern et al. [34], the limit of 400,000 cells/mL would be the most suitable one for detecting problems of mastitis in meat sheep breeds. On the other hand, Olechnowicz and Jaśkowski [1] suggested that in dairy sheep, SCC between 200,000 and 400,000 cells/mL indicates subclinical mastitis.

Another relevant diagnostic tool is microbiological culture, which aims to isolate the pathogen causing the infection. Consequently, the milk sample should be incubated in a culture medium and checked for colony growth. Further identification of bacteria is also required, which is time-consuming. This method has disadvantages; particularly the length of time it takes for testing as well as the frequent incidence of ‘no growth’ cases in mastitis milk cultures. Other diagnostic tools and markers of mastitis in dairy sheep include collecting samples from the udder tissue of infected ewes and subsequently performing histological and immunohistochemical analyses have been described, but concurrently impractical and implemented mostly in the research studies [35]. 

However, all these tests have their shortcomings that necessitate the introduction and development of more sensitive and reliable predictors of mastitis. An increase in SCC and positive bacteriology for mastitis pathogens in milk samples are indicative of subclinical mastitis but the evidence of only one of these alterations must suggest an uncertain case of subclinical mastitis [28]. Unfortunately, animals with SM often remain untreated because the disease may not be revealed and this creates a real thread for animal health, farm income, and public health. Nevertheless, SCC and bacteriological examination are expensive, time and labor-consuming, and are not yet in use at the farm level in dairy ewes. Therefore, researchers are looking for a new promising diagnostic tool as following the world-recognized mastitis expert, who claims that investments in defining mastitis control strategies for minor dairy species (such as dairy sheep, goats, and buffalo) are needed [36].

There are many reports in the literature confirming that genetics has a significant impact on mastitis control and diagnosis in ewes [37]. The effect of allelic polymorphism of different genes on SCC has been demonstrated. For example, Świderek et al. [38] established significant differences between SCC level and the percentage of CD4, CD8, and CD19 lymphocytes in the milk depending on the alleles of the Ovar-MHC genes (OLADRB1, OLADRB2, OMHC1). In sheep milk contained <200 × 10^3^/mL SC, they indicated 488 bp (DRB1) and 284 bp (DRB2) more frequently. However, in milk contained >200 × 10^3^/mL somatic cells, they detected 508 bp (DRB1) and 272 (DRB2) alleles more often. Additionally, Sutera et al. [39] identified few candidate genes associated with SCC in Valle del Belice sheep related to immunity system and udder conformation. They detected eight significant SNPs (single nucleotide polymorphisms) for SCC located in five different chromosomes, and among these, only one marker reached the genome-wide significance threshold. The most significant SNP associated with SCC is located in the region of SERP_1_ (stress-associated endoplasmic reticulum protein 1). Moreover, these results suggest that individuals with the GG genotypes at rs401598547, CC at rs161717499, and AA at rs403091159, rs422960374, and rs426621433, could be selected to reduce the content of the somatic cell in milk [39]. On the other hand, research conducted by Banos et al. [40] confirmed the presence of animal genetic variability in mastitis resistance and identified genomic regions associated with specific mastitis traits in the Chios sheep. For this research, they genotyped 609 ewes with a custom-made 960-single nucleotide polymorphism DNA array based on markers located in quantitative trait loci (QTL) regions for mastitis resistance. SNP markers located in 5 chromosomes and relevant candidate genes implicated in innate immunity (SOCS_2_, CTLA_4_, C6, C7, C9, PTGER_4_, DAB_2_, CARD6, OSMR, PLXNC_1_, IDH_1_, ICOS, FYB, and LYFR) were indicated [40]. 

All of these studies confirmed that searching for and learning about new genetic polymorphisms will facilitate the diagnosis and prevention of mastitis, and also will indicate new directions for breeding work including genetic selection. There is a need to consider genetic improvement for reduced susceptibility to mastitis, as a sustainable means to control the disease. However, selection for resistance to mastitis is difficult because of its polygenic nature, where many genes with small effects are involved [21].

## 5. Promising Novel Inflammatory Markers of Mastitis in Sheep

A permanent search is underway for other indicators of inflammation that would enable more efficient, sensitive, and specific detection of mastitis. These indicators should constitute an alternative to SCC or as a supplement for evaluation or improving SCC performance [41,42]. Researchers should look for the molecules that are released into the milk by the mammary gland during inflammation. This indicator should be a molecule, enzyme, or protein that is practical for detection with enzymatic assays or other immunoassay procedures [41]. Recent investigations and data carried over from humans, as well as veterinary medicine, show that acute-phase proteins (APP), microRNAs (miRNAs; short, non-coding RNAs), and cathelicidins measurement may be the tools needed to improve the early diagnostics of ovine mastitis [43,44,45].

## 6. Acute-Phase Proteins

Acute-phase response (APR) is characterized by several systemic reactions, including fever, catabolism of muscle protein, alterations in sleep and appetite patterns, and changes in the concentrations of a group of serum proteins called acute-phase proteins (APP) [46,47]. The APPs have been empirically defined as proteins whose plasma concentration increase (the positive APPs) or decrease (the negative APPs) by 25% or more following an inflammatory stimulus [47,48]. APPs are induced by pro-inflammatory cytokines such as interleukin 1 (IL-1), IL-6, and tumor necrosis factor alfa (TNF-α). Cytokines are released from the inflammatory site into the circulation in waves of relatively short duration and are supplemented by a paracrine production of the same cytokines by the hepatic Kupffer cells [49]. The role of the acute phase is to restore the disturbed physiological processes of homeostasis. The response of APP, as a kind of reactive protein and as the degree of change, is different in companion animals, livestock, and humans as shown in previous studies [47]. Transferrin is known to be a negative APP in most species of mammals, but in chickens, transferrin reacts as a positive protein [50]. Furthermore, the concentration of each APP can increase to varying degrees depending on the causative agent of the acute phase response. Haptoglobin (Hp) is a strongly reacting acute phase protein in most species studied, including man, mouse, rat, pig, cattle, and rabbit [51]. While CRP (C-reactive protein) is reported not to be an APP in cattle [52] but increases 7–10 times during the acute phase response in the pig [53,54,55]. SAA (serum amyloid A) is an acute-phase protein also in cattle, increasing by over 5 times during the acute phase [43].

Haptoglobin, likewise in cattle, is an important APP in sheep [56]. With SAA, it is considered a major APP in sheep. It is known in many cases to be a post-acute marker of the inflammatory process. Several functional properties of Hp have been described. The major biologic function of Hp is to bind hemoglobin in an equimolar ratio with a very high affinity to prevent hemoglobin-mediated renal parenchymal injury and loss of iron following intravascular hemolysis. The level of serum Hp in healthy sheep is highly variable but most authors point to a physiological value below 0.3 mg/mL, while a Hp above 1 mg/mL is considered the approximate cut-off of severe inflammation [56].

SAA proteins comprise a family of apolipoproteins coded for by at least three genes with allelic variation and a high degree of homology between species [57]. The synthesis of certain members of the family is greatly increased in inflammation. SAA proteins can be considered apolipoproteins as they associate with plasma lipoproteins mainly within the high-density range, perhaps through the amphipathic alpha-helical structure. The physiological role of SAA in the host defense during inflammation is not well understood, but various effects have been reported [46]. These include the inhibition of lymphocyte proliferation, detoxification of endotoxin, inhibition of platelet aggregation, inhibition of thrombocytes aggregation, and inhibition of oxidative reaction in neutrophils [46,58]. It is important to note that there is an extrahepatic synthesis of a specific isoform of serum amyloid A directly from mammary epithelial cells.

Alpha-1 acid glycoprotein (AGP) is a highly glycosylated protein synthesized mainly by the liver but extrahepatic production (notably epithelial and endothelial cells) has also been described [46,59]. AGP is considered a moderate APP in sheep. Like serum albumin, AGP is a binding protein in plasma [60]. In physiological conditions, AGP can bind more than 300 different biologically active endogenous and exogenous substances such as heparin, histamine, serotonin, steroids, catecholamines, and drugs [59,61,62,63]. Recently, AGP has been identified in milk and mammary tissue in cattle, as a potential biomarker of mammary inflammation; however, research is currently limited. 

In modern laboratory diagnostics, special attention is paid to acute-phase proteins as an indicator of mammary gland inflammation in cows and sows [43,64,65,66]. It is important to note that APP might be also determined in milk, which makes sampling easier and less harmful for the animals [65]. Studies conducted to date have demonstrated that APPs concentrations in cows with mastitis are reliable as diagnostic biomarkers. For example, SAA, which is displaying multiple isoforms in plasma and different body fluids including mammary secretion (milk amyloid A—MAA), has been investigated as a marker of mastitis in cows [64,65]. Moreover, APPs can be used as an auxiliary tool in the diagnosis of viral diseases in ruminants [67]. 

In sheep, compared to other species (cattle, pigs) APP profile analysis has not been the subject of such intense research to date, however, a significant increase is observed in this area during the last decade [67,68,69]. In these species of animals, the most positive APPs are Hp and SAA. AGP is considered as a moderately positive APP, while fibrinogen and ceruloplasmin as minor positive APP. Negative APPs in sheep include albumin [68]. APPs ranges in healthy sheep have been published previously [70,71]. 

Regarding ovine mastitis, to our knowledge, there are only two studies investigating APP response in case of naturally occurring mastitis [28,72], but the studies are not complex, since they investigated the APP concentration only in one diagnostic matrix, Simplicio et al. [72] in serum, while Miglio et al. [28] in milk. There is also a complex study investigating the protein profile changes in ewes experimentally infected with *Mannheimia haemolytica* [73], which reveals that after bacterial inoculation the protein profile of blood plasma as well as milk changed significantly. For example, in ewes blood plasma there were identified 33 differentially abundant proteins (compared to findings before the challenge): 6 with decrease, 13 with new appearance, and 14 with varying abundance [73] including acute-phase proteins. Simplicio et al. [72] investigated serum APP concentration in ewes and goats with naturally acquired staphylococcal mastitis and claimed that increase in serum ceruloplasmin, fibrinogen, haptoglobin, and α1-acid glycoprotein was of 337%, 90%, 461%, and 225%, respectively. While Miglio et al. [28] reported the highest milk MAA concentration in ewes with subclinical mastitis comparing to healthy ewes (114.37 ± 41.14 vs. 29.68 ± 27.98 μg/mL). The growing importance of APP as diagnostic markers of mastitis may be associated with the potential of these proteins to not only signal the presence of mastitis but also the specific causative pathogen, reliably. Changes in APP serum concentration in sheep have been also studied during infections or other pathological conditions [69,74,75]. For example, El-Deeb et al. [69] examined APP concentration in sheep with *Coxiella burnetii* infection-induced abortion or sheep with pneumonic pasteurellosis [76] and concluded that determination of APP concentration can be an auxiliary tool in diagnostics of these diseases. On the other hand, Meling et al. [75] described an acute phase response in sheep with clinical scrapie, while Sanchez-Cordon et al. [74] determined acute phase response in the case of both experimentally and naturally occurring bluetongue and found a correlation between APP and the evolution of clinical signs and gross lesions.

## 7. miRNAs

Since their accidental discovery in nematodes, microRNAs (miRNAs) have emerged as key regulators of biological processes in animals [77]. MicroRNAs are short, non-coding RNAs, 18–25 nucleotides in length [78], which are known to regulate biological processes. They are responsible for controlling the expression of protein-coding genes and participate in the regulation of many cellular processes in animals; miRNAs regulate gene expression by inhibiting translation initiation or elongation and inducing co-translational protein degradation and premature termination of translation [79]. It includes immune system activity, which in turn makes miRNAs having specific roles in disease and inflammation pathogenesis. Consequently, it opens new promising perspectives in diseases diagnostics. miRNAs have been investigated as non-invasive biomarkers of various diseases in humans, including cancer, cardiac disease, infection, and inflammatory disease [80]. 

In farm animal diseases, the miRNA’s utility as a biomarker has been recently reviewed [79]. The authors point out that in cattle miRNA can be used in diagnostics (as well as management) of Johne’s diseases (JD), bovine viral diarrhea (BVD), and mastitis, while in pigs, miRNAs have been investigated in the case of the porcine reproductive and respiratory syndrome (PRRS), swine influenza and, to a lesser extent, salmonellosis, adenomatosis and colibacillosis [79]. In poultry diseases, the literature describes miRNAs shifts in Marek disease, avian leukosis, bursal disease, and avian influenza [79]. There are also reports from equine veterinary medicine, that miRNAs can be useful diagnostic tools in different pathologies in horses including osteochondrosis, myopathies, and infertility [81]. Regarding sheep, miRNA has been investigated for assessing carcass quality [82], determining nutritional status before breeding [83], or the sheep were used as a model for human medicine in heart diseases [84]. There are also papers regarding miRNA dynamics in experimentally induced LPS-challenge [85], blue-tongue infection [86], or cystic echinococcosis [87]. miRNAs have been also investigated as a marker of milk and protein yield [88] or their concentration has been described during mammary gland development [78] or during late gestation and the early neonatal period in cardiopulmonary tissues, suggesting their important role in the fetal development [89]. Generally, miRNAs are reported to be very sensitive and specific. miRNAs are present in many tissue and body fluids including blood, mammary secretion and mammary gland parenchyma [90], and the urine of animals. miRNA have been identified in the milk of cows, pigs, humans, goats, sheep, rats, and yaks [80]. It is important to highlight that miRNA in milk are stable and resistant to acidic environments, RNase digestion, incubation at room temperature, and multiple freeze/thaw cycles. This suggests that miRNA in milk could potentially be used as a biomarker or for milk quality control. It is of special importance since milk samples can be obtained during routine milking, causing no harm to the animal. Recent studies revealed twenty-five bovine mi-RNAs differentially expressed during mastitis relative to their expression in normal milk [91]. Special attention was paid to miR21, miR146, miR383, and miR92a as a reference molecule as these bovine miRNAs exhibit sensitivity and specificity greater than 80% for differentiating between California Mastitis Test positive (CMT+) milk and normal milk [80]. On the other hand, selected serum bovine miRNA can be also used as a mastitis biomarker suggesting the impact of local inflammation on the systemic reaction, but the sensitivity and sensibility are inferior compared to bovine milk miRNA concentration. Bovine miRNAs concentration can already be used as a diagnostic tool, for example, Srikok et al. [44] claimed that bovine milk miR29b-2, when used in combination with the CMT and days in milk data, was applicable for screening and classification of milk samples from cows as healthy, subclinical mastitis, or mastitis. Interestingly, there are also reports suggesting that bovine miRNAs expression pattern can be pathogen-unique as described by Ngo et al. [92], who identified 27 miRNAs unique to *Streptococcus uberis* mastitis with an emphasis on miR-320a and miR-320b due to their roles in the modulation of trained immune activity. The reports suggest that miRNA profiles might be pathogen-specific demonstrating unique miRNA biomarkers depending on bacteria species. However, these are most in vitro studies performed on bovine epithelial cells [79].

The majority of studies regarding milk miRNAs concentration and mastitis are carried out in cattle. So far there is a lack of data regarding miRNA expression in milk during naturally occurring ovine mastitis, thus research in this matter is highly desired [92]. However, based on the similarities between these two species of ruminants, it is highly probable that milk miRNAs concentration will be in the future sensitive biomarker of ovine mastitis.

Furthermore, as aforementioned from a genetic point of view genotyping and determining which ewes are genetically predisposed to mastitis can be used as part of long-term diagnostics and mastitis control, as it was proven that the susceptibility of dairy sheep to udder infections is heritable. Basing breed selection programs on animals with genetically superior mastitis resistance could contribute to increased breeding progress [40].

## 8. Other Non-Coding RNAs

An important role in many biological processes is played by non-coding RNAs (ncRNAs) sequences that regulate gene expression after transcription [88]. Special attention should be drawn to long non-coding RNAs (lncRNAs), which are more tissue-specific and are distinguished from other ncRNAs based on their large size (longer than 200 nucleotides) [93]. There is a growing number of reports on their involvement in host cell response (proliferation, differentiation, and apoptosis) to bacterial infections [73], as they are involved in epigenetic regulation, chromatin organization, transcriptional control, and pre- and posttranslational mRNA processing [93,94]. In addition, there have been reports about a novel mode of action of lncRNAs as competitive endogenous RNAs (ceRNAs), which affect the expression of mRNAs [95].

Tong et al. [94] in their study indicated that lncRNAs were involved in bovine susceptibility to CM, milk yield and quality and Wang et al. [96] established a bovine mastitis cell model, which they used to determine whether the lncRNAs participate in the progression of mastitis. It has been stated that the lncRNAs influence inflammation cascades and therefore their dysregulation could become a biomarker of a bovine mammary-gland inflammation. Although Hao et al. [97] studied the profiles of lncRNAs in the mammary gland from ewes and revealed their comprehensive expression to better understand their functions in ovine lactation, there is still a small number of reports on the roles of lncRNAs in ovine mammary gland tissues. As Do et al. [79] noticed, there is a downward trend in the sequencing expenses, which may in the future lead to creating more opportunities for sequencing multiple types of molecules such as ncRNAs, miRNAs, mRNAs, and others. This may contribute to increased detection and a better understanding of mastitis pathogenesis among small ruminants, as well as selection and prediction of treatment response. Therefore, non-coding RNAs and long non-coding RNAs should be considered as a future perspective for a potential mastitis biomarker [79].

## 9. Cathelicidins

Cathelicidins (CATH) are among the most promising molecules for mastitis detection in sheep [98]. CATH is one of many proteins that can be tested by proteomics research methods including MALDI-TOF MS. In the future, proteomics probably would be used to improve the elucidation of mastitis pathogenesis [45]. The term “cathelicidins” was coined in 1995 from cathelin, because of the characteristic cathelin-like domain present in these proteins [99]. Cathelicidins are part of the innate immune system of many vertebrates, e.g., humans and farm animals (cattle, horses, pigs, sheep, goats, chickens, rabbits, and fish), that have numerous functions, essentially direct antimicrobial activity (against bacteria, enveloped viruses, and fungi), also pro-inflammatory and chemotactic functions [100,101]. This group of proteolytically activated peptides is derived from 8 genes [101], including cathelicidin-1, -2, and -3 that are expressed in milk during mastitis [102] also cathelicidin-derived myeloid antimicrobial peptide [98]. A significant amount of cathelicidins is present in milk leukocytes [103] and the main producers of CATH are neutrophils [104]. These molecules are massively released from neutrophils in response to microbial stimulus, always before clinical signs of mastitis [105]. The second source of cathelicidins are mammary epithelial cells (MECs), which release these molecules in response to the entrance of pathogens [98,105]. Cubeddu et al. [104] confirmed that MECs release cathelicidins before leukocyte influx in the milk. Cathelicidins from both sources constitute the first line of defense in the mammary gland against pathogenic microorganisms, but CATH from neutrophils is the main source of these proteins in milk. Addis et al. [106] confirmed that the measurement of cathelicidins in milk by ELISA provides extra sensitivity while maintaining high specificity. Therefore, investigation of cathelicidins in milk with simultaneous SCC improves detection of subclinical mastitis. Puggioni et al. [107] evaluated SCC and CATH in late lactation sheep milk for their relationship with intramammary infections. They demonstrated that CATH has a higher specificity than SCC (82.92% vs. 73.67%), but both have similar sensitivity, total about 91.8%. Therefore, in late lactation ewes, CATH is a more desirable method to indicate intramammary infections. Katsafadou et al. [73] confirmed that detection of cathelicidin-1 in sheep milk is significantly associated with the presence of mastitis in ewes. This association is the strongest in the first 24 h after infection. A positive CATH-1 testing result is sufficient for the diagnosis of mastitis in ewes. Along with the progressive antibiotics’ resistance in many commonly encountered bacteria, CATHs are potential new bactericidal agents with therapeutic capability in persons and individuals with cystic fibrosis [108,109]. In vitro studies conducted by Brogden et al. [110] demonstrate that the administration of CATH to lambs with pneumonia reduces the concentration of bacteria in bronchoalveolar lavage fluid and consolidated pulmonary tissues. Moreover, cathelicidins have broad-spectrum antimicrobial activity against all isolates of *Pasteurella multocida* [111], also against spirochaetes [112]. These studies indicate the utility of CATH in the treatment of respiratory tract infections, but in the future, can help in the treatment and/or diagnostics of other biological systems including the mammary gland. 

The brief characteristics of potential novel biomarkers of ovine mastitis could be found in Table 2.

## 10. Summary

Mastitis is a complex and severe disease in ewes causing significant losses to the dairy industry. It is important to note that its management depends mainly on diagnostics. Therefore, novel biomarkers with higher sensitivity and accuracy are highly desired in this matter. These include acute-phase proteins, miRNAs, and/or cathelicidins. Currently, these markers are under intensive research, but in the future, they could potentially be applied in routine veterinary diagnostics.

It is important to highlight that application of reviewed biomarkers is currently limited. Up to date the combination of bacteriological and cytological examinations is considered to be the most reliable means of diagnosing ovine subclinical mastitis. Although, they are not as widely used as in dairy cattle. The limitations of reviewed potential novel biomarkers may have different causes. On the one hand, the cost of laboratory analyses seems to be relatively high (e.g., specialized equipment is needed for proteomic or miRNAs analyses). In particular, taking into account the sheep and its milk economic value. On the other hand, the proposed biomarkers in sheep are currently under intensive research and their role is not fully understood. Therefore, further studies are required to assess their utility as diagnostics tools in ovine mastitis. In the future probably, if the cost of proposed biomarkers laboratory analyzes would become lower, then they would gain significance.

## Figures and Tables

**Table 1:** Prevalence of subclinical ovine mastitis in different management systems.

Management System	Prevalence of Subclinical Mastitis	References
Semi-intensive	0.296	[21]
	0.120	[22]
	0.112	[23]
Intensive	0.254	[21]
Semi-extensive	0.196	[21]
	0.192	[24]
	0.139	[25]
Extensive	0.178	[21]
	0.192	[24]

**Table 2:** Brief characteristics of new potential biomarkers for mastitis diagnosis in sheep.

Type of Biomarker	Mode of Action	Brief Description	Current State of Action	Concentration Changes	Key Reference
Acute-phase proteins (APP)	- restore the disturbed physiological processes of the homeostasis	- induced by pro-inflammatory cytokines, - haptoglobin, serum amyloid A (SAA), and its milk isoform (MAA), alpha-1 acid glycoprotein (AGP)	- indicator of mastitis caused by S. aureus in goats and sheep - diagnosis of viral diseases in ruminants	- staphylococcal mastitis: increase serum ceruloplasmin by 337%, fibrinogen by 90%, haptoglobin by 461%, and alpha-1 acid glycoprotein by 225%	[72]
miRNA	- control the expression of protein-coding genes (inhibition, elongation, degradation, termination)	- short, non-coding RNAs - regulate many cellular processes - play role in disease and inflammation pathogenesis - occur in many tissue and body fluids	- non-invasive biomarkers of various diseases - very sensitive and specific - in milk be used as biomarkers or for milk quality control	- 25 mi-RNAs differentially expressed during mastitis relative to their expression in normal milk - lack of data regarding miRNA expression in milk during naturally occurring ovine mastitis	[79]
Cathelicidins (CATH)	- direct antimicrobial activity - proinflammatory and chemotactic functions - released from neutrophils	- proteolytically activated peptide, - express in milk during mastitis - cathelicidin-1, -2, -3 and cathelicidin-derived myeloid antimicrobial peptide - the first line of defense in the mammary gland	- most promising molecules for mastitis detection in sheep - bonus sensitivity with high specificity - has a higher specificity than SCC and similar sensitivity	- detection of cathelicidin-1 in sheep milk confirms mastitis	[107]

## Data Availability

Not applicable.
